# Human Exposure Assessment of Indoor Dust: Importance of Particle Size and Spatial Position

**DOI:** 10.1289/ehp.1206470

**Published:** 2013-04-01

**Authors:** Zhiguo Cao, Gang Yu, Bin Wang, Jun Huang, Shubo Deng

**Affiliations:** POPs Research Center, School of Environment, State Key Joint Laboratory of Environment Simulation and Pollution Control, Tsinghua University, Beijing, China, E-mail: yg-den@mail.tsinghua.edu.cn

Stapleton et al. (2012) reported that

serum ΣpentaBDEs [sum of pentabrominated diphenyl ethers 47, 99, 100, and 153] were significantly correlated with both handwipe and house dust ΣpentaBDE levels, but were more strongly associated with handwipe levels (*r* = 0.57; *p* < 0.001 vs. *r* = 0.35; *p* < 0.01).

Here we propose an explanation for this phenomenon.

Toxicants are not distributed homogeneously in dust according to particle size; particle size distribution of settled dust varies with its spatial position. Thus, distribution of polybrominated diphenyl ethers (PBDEs) will vary with the particle size of dust and the spatial position of settled dust, as well as the location of PBDE sources, such that PBDE levels in settled dust on the floor will be different from those of settled dust above the floor (Björklund et al. 2012; Wu et al. 2010). Because of the spatial position of particles, humans are likely to be exposed only to particles of specific sizes. In addition, exposures to children and adults may be different because particles to which children and adults are exposed often have different spatial position and particle size distribution (Cao et al. 2012; Ruby and Lowney 2012). In addition, the reliability of human exposure assessments may be substantially influenced by between-room and within-room spatial variability of PBDE concentrations in indoor dust (Muenhor and Harrad 2012).

As reported in many other studies, Stapleton et al. (2012) reported that for dust sampling, they vacuumed “the equivalent of the entire floor-surface area for the room … by gently drawing the crevice tool across the top of all surfaces,” and they selected fractions < 500 µm for their analysis. Only a few studies have demonstrated that particles > 250 µm are not appropriate for risk assessment of human exposures (Cao et al. 2012). Thus, if the dust samples from the house and from handwipes have different particle size distributions and are from different spatial positions in the indoor environment, it is inevitable that the PBDE levels in handwipes and house dust will be different and the correlation between PBDE in human serum and house dust will be weaker.

For human exposure assessment, we propose that indoor dust samples to be analyzed should be from relevant spatial positions and of specific particle size. By improving sampling strategies, we can obtain more accurate results and the correlations between PBDEs in human tissues and indoor dust will be much more accurate. In addition, settled dust should be sampled separately for adults and children.
